# Eye-mask and earplugs as sleep aid on glucose metabolism in sleep-deprived pregnancy: a randomized controlled trial

**DOI:** 10.1186/s12884-026-09283-y

**Published:** 2026-06-26

**Authors:** Ahmad Sani Nurulhuda, Normala Mohammad Som, Jesrine Hong, Ik Hui Teo, Annamalai Vimaladevi, Farah Gan, Mukhri Hamdan, Peng Chiong Tan

**Affiliations:** 1 Department of Obstetrics and Gynecology, Faculty of Medicine, Universiti Malaya, Jalan Profesor Diraja Ungku Aziz, Kuala Lumpur, 50603 Malaysia; 2 Department of Obstetrics and Gynecology, Faculty of Medicine, Universiti Teknologi MARA (UiTM), Jalan Hospital , Sungai Buloh, Selangor 47000 Malaysia

**Keywords:** Sleep, pregnancy, Eye-mask, Earplugs, Actigraphy, Glucose, Gestational diabetes

## Abstract

**Background:**

Gestational diabetes is associated with short sleep duration. Both conditions are common in pregnancy, especially in later pregnancy. Eye mask and earplugs when used at home in sleep-deprived pregnancy has been shown to extend nocturnal sleep. Data is sparse on the effect of sleep aids on glucose metabolism through extending sleep. We sought to evaluate use of eye-mask and earplugs on fasting and 2-hour plasma glucose levels at routine oral glucose tolerance test (OGTT) and on actigraph-derived night sleep duration in women with sleep-deprived pregnancy.

**Methods:**

A randomized trial was conducted in a university hospital in Malaysia from July 2021 to August 2022. Sleep-deprived (≤ 7 h night sleep duration) women, on being scheduled for routine OGTT for gestational diabetes screening were enrolled. Participants were randomized to the use of eye-mask and earplugs as sleep aid or no intervention in the week prior to OGTT appointment. All participants were asked to wear the actigraph watch during night sleep in the week prior to OGTT. Pittsburgh Sleep Quality Index (PSQI) data was also obtained at enrollment and at OGTT. The primary outcome was fasting and 2-hour glucose levels at OGTT and actigraph-derived night sleep duration. Secondary outcomes were actigraph-derived wakening after sleep onset, sleep efficiency, and PSQI-derived sleep quality and self-reported night sleep duration. The t test, Mann-Whitney U test, Chi square test, and Fisher exact test were used as appropriate for the data.

**Results:**

240 women were randomized; 120 to each intervention. Data on OGTT results were available from 116 to 117 participants and on actigraph-derived sleep data from 100 to 103 participants for eye-mask and earplugs vs. no intervention arms respectively. The primary outcome of fasting glucose level was mean ± standard deviation (mmol/l ) 4.4 ± 0.5 vs. 4.3 ± 0.4, mean difference (95% CI) 0.03 (-0.86 to 1.46) *p* = 0.611, 2-hour level was 6.3 ± 1.4 vs. 6.4 ± 1.3, mean difference (95% CI) -0.11 (-0.45 to 0.23) *p* = 0.536, and total night sleep duration (minutes) 347 ± 50 vs. 349 ± 47, mean difference (95% CI) -2.6 (-16.0 to 10.9) *p* = 0.707for eye-mask and earplugs vs. no intervention arms respectively. Secondary outcomes were also not significantly different.

**Conclusion:**

One-week use of eye-mask and earplugs as a sleep aid in women with sleep-deprived pregnancy does not decrease glucose levels at oral glucose tolerance test or extend actigraph-derived sleep duration.

**Ethics oversight:**

This trial was approved by the Medical Research Ethics Committee of University Malaya Medical Centre (UMMC) on July 1, 2021 (reference number MREC ID NO: 202157 − 10120).

**Clinical trial registration:**

This study was registered in ISRCTN registry on July 14, 2021, with trial identification number: ISRCTN 17,526,076, http://www.isrctn.com/ISRCTN17526076.

**Supplementary Information:**

The online version contains supplementary material available at https://doi.org/10.1186/s12884-026-09283-y.

## Introduction

As pregnancy advances, sleep duration decreases, wakening and length of time awake increase, and overall sleep quality diminishes [1]. Almost 50% of pregnant women experience short sleep by term [2]. It is recommended that adults, including pregnant women, obtain 7–9 h of sleep per night [3]. In nulliparous women with a singleton pregnancy, median sleep duration was 7.4 h; approximately 27.9% had a sleep duration of < 7 h and 2.6% had a sleep duration of > 9 h [4].

The prevalence of gestational diabetes mellitus (GDM) is increasing [5, 6]. In 2017, 16.2% of live births were impacted by hyperglycemia in pregnancy with GDM accounting for 86.4% of these cases according to the International Diabetes Federation Diabetes Atlas [7]. The observed increase in incidence of gestational diabetes was attributable to changes in screening practices (primarily changes in screening methods) rather than changing population factors [8].

Short sleep duration in pregnancy increases the risk of developing GDM [9]. Short (< 7 h) or long sleep (≥ 9 h) in early pregnancy were associated with GDM [10] indicating a U-shape risk relationship [11].

In the hospital setting such as in the intensive and high dependency care, post-surgery care, and environments with persistent light and sound stimulation, eye-mask and earplugs (EMEP) are proven as sensory deprivation sleep aids, prolonging sleep duration by up to 40% [12–14]. Use of EMEP in the bedroom environment at home in sleep-deprived pregnancy extends night time sleep by 23–25 min [15–17]. To our knowledge, no prior studies in either pregnant or non-pregnant adults have evaluated the effects of EMEP interventions on glucose metabolism. Previous trials involving EMEP have focused primarily on improving sleep duration and quality, both in hospital and home settings [12–17]. This study therefore represents the first proof-of-concept investigation into whether sensory deprivation during sleep could influence maternal glucose regulation.

We hypothesized that in women with short sleep duration (< 7 h), EMEP use during night sleep in the week prior to their routine GDM screening with the 75-g oral glucose tolerance test (OGTT) would reduce their fasting and 2-hour glucose levels in a proof-of-concept randomized trial.

## Materials and methods

The study was designed as a parallel, randomized controlled trial. Women aged 18–40 years old were recruited at the antenatal clinic as they were given their appointment for OGTT for routine GDM screening. All women in our center were offered GDM screening with the 75 g OGTT in a two-stage process. Screening was performed in high-risk women at booking for antenatal care in our center and to be repeated at about 28 weeks if GDM screen was negative, and in all others at 24–28 weeks [18]. Eligibility was assessed using the eligibility assessment form (EAF) after the medical records of women offered GDM screening were scrutinized.

Trial inclusion criteria were women with self-reported night sleep of ≤ 7 h, singleton pregnancy with gestational age ≤ 34 weeks. Those who had prior OGTT, pre-existing sleep, psychiatric or medical disorders, active smokers, current alcohol consumption, body mass index (BMI) above 35 kg/m^2^, multipara with co-sleeping child/children, night caretaker of other family members, night shift worker, disinclination to use EMEP or ActiGraph device, known gross fetal anomalies, or intrauterine death were excluded. Women with BMI > 35 kg/m² were excluded to reduce confounding from obesity-related metabolic alterations and higher baseline risk of GDM, which could mask or exaggerate the modest metabolic effect expected from a sleep intervention. Similar rationale applied to other exclusions (e.g., night-shift work, alcohol intake, psychiatric disorders), which could independently affect sleep and glucose metabolism.

The Pittsburgh Sleep Quality Index (PSQI) questionnaire was provided. A stem answer to the PSQI was used to derive self-reported sleep duration in whole hours. Potentially eligible participants were given the Participant Information Sheet (PIS), and the recruiting investigator encouraged and responded to their queries. All participants provided written informed consent.

The randomization sequence was generated in blocks of 4 or 8 by a co-investigator who was not involved in the recruitment using a random sequence generator provided by random.org in a 1 to 1 ratio. Randomization was implemented by opening the lowest-numbered sealed envelope still available to the most recent recruit in strict sequential order. Participants were randomized to EMEP (intervention group) or no intervention (control group).

In the EMEP arm, the participant was provided with a sleep diary, EMEP, and the ActiGraph wGT3X-BT device (ActiGraph, Pensacola, FL, USA) and taught on its usage. The actigraphy device was to be worn like a wristwatch, fitting snuggly against the wrist, and not be allowed to wobble around. EMEP were to be used for seven consecutive nights prior to OGTT when in bed for sleep, along with the actigraph device. EMEP could be removed when participants mobilized during the night but were to be re-worn on returning to bed to sleep. The EMEP and the actigraph device were removed on morning awakening. Two sets of EMEP were provided without charge to participants randomized to the intervention arm. They could be reused by the participant until no longer fit for their purpose.

In the no intervention control arm, participants were provided with sleep diary and an identical actigraph device. The same instruction on actigraph device use was given.

### Trial instruments

#### Pittsburgh sleep quality index (PSQI)

The PSQI questionnaire is a well-validated and widely used 9-item self-reported instrument for measuring adult sleep quality and disturbances. A global score of > 5 indicates a “poor sleeper,” with a diagnostic sensitivity of 89.6% and a specificity of 86.5% for persons with primary insomnia [19]. The PSQI questionnaire has been used and validated in pregnancy [20–22].

Participants answered the questionnaire at recruitment and again at the OGTT (modified to assess symptoms in the last week only instead of last month). PSQI data were entered into a Case Report Form and checked for completeness. Global PSQI scores were computed following standard scoring instructions. If a participant omitted any item within a component, the component was treated as missing. Participants with > 2 missing components were excluded from the PSQI-derived analyses. For those with ≤ 2 missing components, mean imputation of the completed items within that component was used. The overall rate of missing PSQI data was < 5%. Analyses were conducted on available data without further imputation.

#### ActiGraph wristwatch

All participants were provided with the device and instructed on its use. Participants were also provided with a diary to keep a daily sleep log to record time in bed and time out of bed for sleep. These data were needed by the ActiLife software (ActiGraph, Pensacola, FL, USA) to calculate sleep duration and other metrics. On their return visit after seven days, participants were asked to return their devices. Data were retrieved and analyzed from these devices.

Actigraphy is a non-invasive technique that measures physical activity levels using a portable device, often resembling a watch, and typically worn on the wrist. Recorded data are subjected to a proprietary algorithm that produces estimates of sleep-wake variables. Actigraphy is particularly useful for long-term monitoring of sleep-wake patterns in the natural environment, such as the home setting. Compared to polysomnography (PSG), actigraphy is more convenient, less invasive, less expensive, and can be worn continuously for an extended time. Actigraphy has been validated (against PSG) in multiple populations, with an estimated agreement between PSG and actigraphy ranging from 91 to 93%, and a correlation coefficient of at least 0.85 in healthy individuals [23].

Actigraphy data were downloaded and processed using ActiLife Software version 6.13.4 (ActiGraph LLC, Pensacola, FL, USA). Each participant’s data were visually inspected for compliance and artifacts. Non-wear periods were defined as ≥ 60 min of consecutive zero activity counts and were excluded. Sleep periods were aligned with each participant’s daily sleep diary entries (bedtime and wake time) to improve scoring accuracy. Only nights with both valid actigraphy data and corresponding diary entries were included in the analysis.

#### Oral glucose tolerance test (OGTT)

The 75-g OGTT was performed under standard conditions. Pre-test, women were asked to consume their normal diet in the three days prior to the test appointment morning date and to fast (except for water) from the midnight before [24]. All participants received pregnancy and delivery standard care in accordance with OGTT results on GDM diagnosis.

Due to the obvious nature of the interventions, participants and investigators were not masked to the interventions. However, both arms were given sleep diary and actigraphy device which might be perceived, especially by the control arm, as an intervention of sorts.

Primary outcomes were the impact of one week use of EMEP on fasting plasma glucose and 2-hour plasma glucose level at OGTT, and on actigraphy-derived night sleep duration. Secondary maternal outcomes were actigraphy derived wake after sleep onset (WASO), actigraphy derived sleep efficiency, and satisfactory sleep measured using PSQI.

### Sample size calculations

#### Glucose levels at OGTT (fasting and 2-hour)

From the most recent data available from all 257 women who had their OGTT in our antenatal clinic from December 2020 to March 2021, the standard deviations for their fasting and 2-hour plasma glucose were 0.47 and 1.31 mmol/L respectively.

Assuming 0.2 mmol/l change in fasting blood glucose across trial arms to be the impact size from EMEP vs. control, factoring in standard deviation for fasting glucose of 0.47 for both arms, we used OpenEpi (https://www.openepi.com/SampleSize/SSMean.htm) online sample size calculator with application of alpha of 0.05, power of 80%, a 1:1 ratio across trial arms and t test, a total sample size of 174 (87 in each arm) was required. Assuming 0.5 mmol/l change in 2-hour blood glucose across trial arms to be the impact size from EMEP vs. control, factoring in standard deviation for 2-hour glucose of 1.31 for both arms, we again used OpenEpi with application of alpha of 0.05, power of 80%, a 1:1 ratio across trial arms and t test, a total sample size of 216 (108 in each arm) is needed. The anticipated effect sizes (0.2 mmol/L fasting, 0.5 mmol/L 2-hour glucose) were chosen to reflect clinically meaningful but conservative changes in glucose observed in prior behavioral and lifestyle interventions aimed at improving maternal glycemia in pregnancy [25, 26].

A previous study by Teo et al.^15^ from our center on the use of EMEP in improving night sleep duration in nulliparas in late pregnancy showed an across arm increase of 6.6 min in EMEP compared to sham head band group, mean ± standard deviation of 24.7 ± 14.9 in EMEP vs. 18.1 ± 17.3 min in sham head band. We conservatively assumed that our no-intervention control arm had the “placebo” effect demonstrated by sham headband. Using OpenEpi (http://www.openepi.com/SampleSize/SSMean.htm), applying alpha of 0.05, power of 80%, 1:1 ratio across trial arms and the t test, a total sample size of 188 (94 in each arm) was required. Factoring in a 10% drop-out rate, we plan to recruit a total of 240 (216/0.9) participants, sufficient to cover all three primary outcomes for a suitably powered study.

Data were entered into a statistical software package SPSS (Version 28, IBM, SPSS Statistics). The 1-sample Kolmogorov-Smirnov test used for normal data distribution. Analysis by t-test was used on normally distributed data, the Mann-Whitney U test for non-normally distributed data or ordinal data, and the Chi-Square test categorical datasets for larger than 5 for 2 × 2 (Fisher’s exact test used instead if > 20% of cells had cell size < 5). Two-sided P values were reported and *p* < 0.05 was regarded as significant. Analysis was on an intention-to-treat basis (data as available).

## Results

**Fig. 1:**
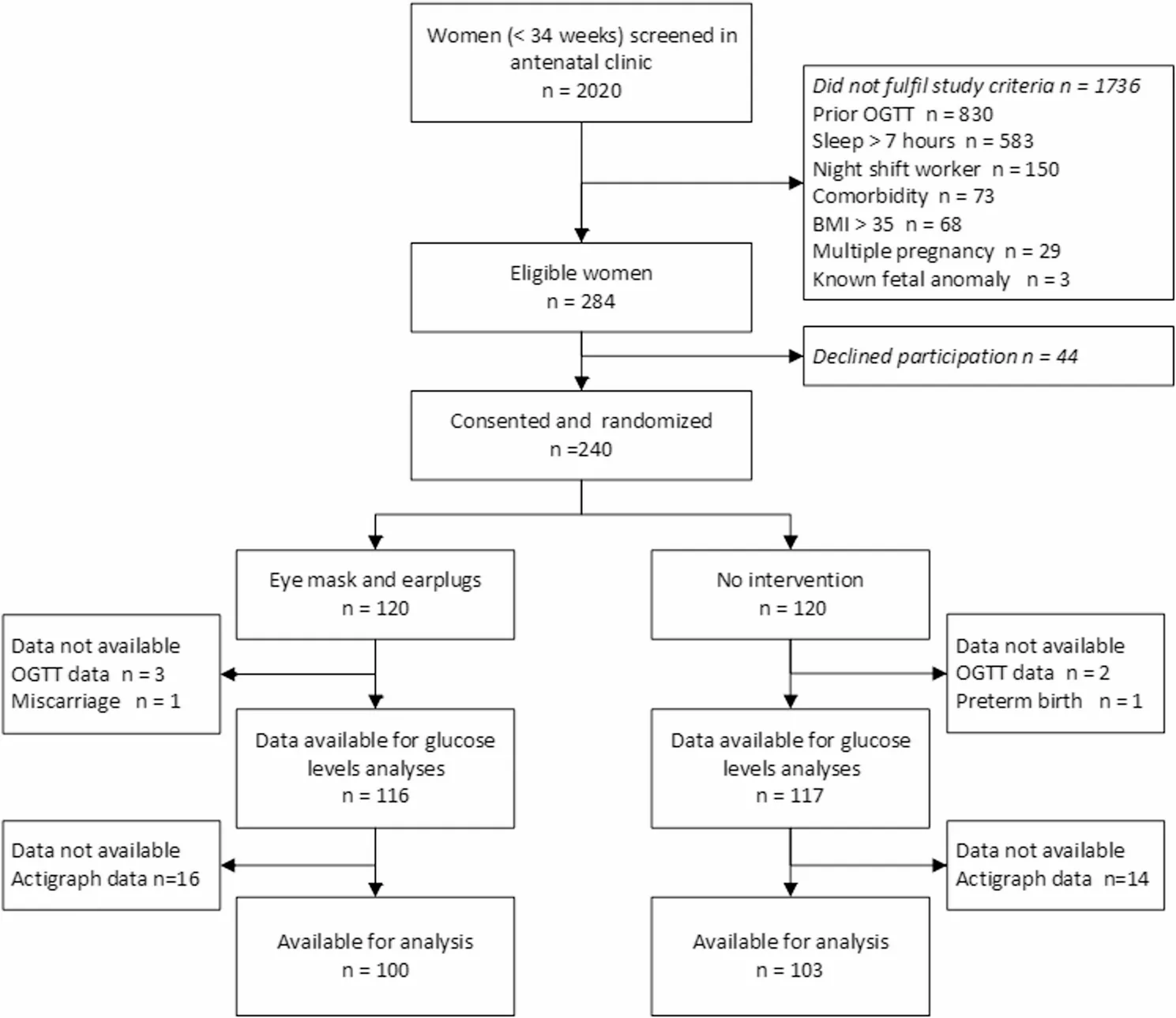
Recruitment flow into a randomized trial of eye-mask and earplugs compared to no intervention in sleep-deprived pregnancy prior to oral glucose tolerance test

Figure 1 depicts the participation flow through the study. The first participant was recruited on July 19, 2021, and the last on August 24, 2022. 2020 women from the antenatal clinic were assessed; 1736 women did not fulfil study criteria (830 had prior OGTT, 583 with sleep duration > 7 h, 150 night-shift workers, 73 with comorbid conditions, 68 had BMI > 35, 29 with multiple pregnancy, and three with gross fetal anomaly). Of the 284 eligible women, 44 declined to participate. 240 women were randomized, 120 to each arm. Recruitment ceased on achieving the target total sample size of 240. For the primary outcomes analyses, OGTT results were not available in seven women (four in EMEP and three in control arm) and a further 30 did not provide any actigraph data (16 in EMEP and 14 in control arm).

Table 1 shows trial participants’ characteristics dichotomized to EMEP or no intervention groups. All recorded baseline characteristics including that of self-reported night sleep duration median[interquartile range] 5.8[5.0–6.0] vs. 6.0[5.0–6.0] *p* = 0.555 and sleep quality at recruitment, BMI, gestational age, and ethnicity were similar, except for participants’ age which was slightly higher in the control arm, 30 [26–33] vs. 31 [27–34] years *p* = 0.026.

**Table 1:** Characteristics of participants with sleep-deprived pregnancy randomized to eye-mask and earplugs (EMEP) or no intervention (control) prior to oral glucose tolerance test (OGTT)

	EMEP *n* = 120	No intervention *n* = 120	*P* value
Age (years)	30 [26–33]	31 [27–34]	0.026
BMI^1^ (kg/m^2^)	25.8 ± 4.3	26.4 ± 4.0	0.316
Gestation (weeks)	24.9 ± 4.6	24.0 ± 4.9	0.135
Ethnicity			0.755
Malay	93 (77.5%)	99 (82.5%)	
Chinese	15 (12.5%)	13 (10.8%)	
Indian	10 (8.3%)	7 (5.8%)	
Others	2 (1.7%)	1 (0.8%)	
Parity			0.196
Nulliparous	63 (52.5%)	53 (44.2%)	
Parous	57 (47.5%)	67 (55.8%)	
Previous history of GDM^2^	3 (2.5%)	11 (9.2%)	0.050
Previous macrosomia	0 (0.0%)	4 (3.3%)	0.122
Family history of diabetes	41 (34.2%)	46 (38.3%)	0.502
Self-reported sleep (hours)	5.8 [5.0–6.0]	6.0 [5.0–6.0]	0.555
	5.5 ± 0.8	5.6 ± 0.8	0.401
PSQI^3^ category			0.421
Poor sleep quality	104 (86.7%)	108 (90.0%)	
Good sleep quality	16 (13.3%)	12 (10.0%)	

Data represented as number (%), mean ± standard deviation, and median [interquartile range]. Analyses were by Student t-test for comparison of means for continuous data, Mann-Whitney U test for non-parametric or ordinal data, Chi-Square test for nominal data (Fisher exact test used instead if > 20% cells have cell size < 4)

^1^Body mass index

^2^Gestational diabetes mellitus

^3^Pittsburgh Sleep Quality Index

Table 2 reports the primary outcomes of fasting and 2-hour glucose levels at OGTT, and actigraph derived night sleep duration. Fasting level was mean±standard deviation (mmol/l) 4.4 ± 0.5 vs. 4.3 ± 0.4 mean difference (95% CI) 0.03 (-0.86 to 1.46) *p* = 0.611, 2-hour level was 6.3 ± 1.4 vs. 6.4 ± 1.3 mean difference 0.11 (-0.45 to 0.23) *p* = 0.536, and total night sleep duration (minutes) 347 ± 50 vs. 349 ± 47 mean difference (95% CI) -2.6 (-16.0 to 10.9) *p* = 0.707.

**Table 2:** Primary outcomes of participants with sleep-deprived pregnancy randomized to eye-mask and earplugs (EMEP) or no intervention (control) prior to oral glucose tolerance test (OGTT)

	EMEP	No intervention	Mean difference (95% CI)	*P* value
	*n* = 116	*n* = 117		
Glucose level at OGTT^1^ (mmol/l)				
Fasting	4.4 ± 0.5	4.3 ± 0.4	0.03 (-0.86 to 1.46)	0.611
2-hour	6.3 ± 1.4	6.4 ± 1.3	-0.11 (-0.45 to 0.23)	0.536
	*n* = 100	*n* = 103		
Total night sleep duration (minutes)	347 ± 50	349 ± 47	-2.6 (-16.0 to 10.9)	0.707

Data represented as mean ± standard deviation. Analyses were by Student t-test for comparison of means for continuous data, Mann-Whitney U test for non-parametric or ordinal data, Chi-Square test for nominal data (Fisher exact test used instead if > 20% cells have cell size < 4)

^1^75-g oral glucose tolerance test

Table 3 illustrates the secondary outcomes. Wake After Sleep Onset (WASO) was 46 ± 39 vs. 40 ± 35 min, mean difference (95% CI) 6.4 (-3.8 to 16.8) *p* = 0.216 and sleep efficiency (%) 88.2 ± 10.0 vs. 89.6 ± 8.8 mean difference (95% CI) -1.6 (-4.1 to 1.0) *p* = 0.236, for EMEP and control arms respectively. When comparing PSQI-derived sleep quality from recruitment to OGTT, both groups demonstrated improvement. The proportion of participants with good sleep quality increased from 13.3% to 24.3% (11.0%) in the EMEP group and from 10.0% to 18.3% (8.3%) in the control group. This suggests a modest improvement over time in both groups, with no clear difference in magnitude between groups (2.7%). Post hoc analyses of PSQI-derived self-reported night sleep duration showed a greater increase in the EMEP group compared to controls. The mean change in self-reported sleep duration was 0.35 ± 0.69 h in the EMEP group versus 0.16 ± 0.78 h in the control group, with a mean difference of 0.20 h (95% CI 0.02–0.38, *p* = 0.030). Consistent findings were observed using the Mann–Whitney U test, with median change of 0 [0–0.5] hours in the EMEP group versus 0 [0–0] hours in the control group (*p* = 0.007). However, the magnitude of improvement in the proportion of participants with good sleep quality appeared similar between groups.

**Table 3:** Secondary outcomes of participants with sleep-deprived pregnancy randomized to eye-mask and earplugs (EMEP) or no intervention (control) prior to oral glucose tolerance test (OGTT)

	EMEP	No intervention	RR (95% CI)	Mean difference (95% CI)	*P* value
	*n* = 100	*n* = 103			
WASO^1^ (minutes)	46 ± 39	40 ± 35		6.4 (-3.8 to 16.8)	0.216
Sleep efficiency (%)	88.2 ± 10.0	89.6 ± 8.8		-1.6 (-4.1 to 1.0)	0.236
PSQI^2^ category	*n* = 113	*n* = 120			
Good sleep quality	28 (24.8%)	22 (18.3%)	1.35 (0.82–2.22)		0.231
Poor sleep quality	85 (75.2%)	98 (81.7%)			
Self-reported sleep (hours)	5.9 ± 0.8	5.7 ± 0.8		0.1 (-0.1 to 0.3)	0.183
Change in sleep (hours)^3^	0.35 ± 0.69	0.16 ± 0.78		0.20 (0.02–0.38)	0.030
	0 [0-0.5]	0 [0–0]			0.007

Data represented as mean ± standard deviation, and median [interquartile range]. Analyses were by Student t-test for comparison of means for continuous data, Mann-Whitney U test for non-parametric or ordinal data, Chi-Square test for nominal data (Fisher exact test used instead if > 20% cells have cell size < 4)

^1^Wakening after sleep onset

^2^Pittsburgh Sleep Quality Index

^3^Change in self-reported sleep duration from at-recruitment to at-oral glucose tolerance test (derived from sleep duration stem question of the Pittsburgh Sleep Quality Index questionnaire)

Analyses were also performed on diagnosis of GDM from the OGTT results based on Malaysian [18], WHO/IADPSG [27], and NICE UK [28] criteria for GDM. According to the various diagnostic criteria, GDM rates were, Malaysia 22/116 (19%) vs. 16/117 (14%) RR (95% CI) 1.4 (0.8–2.5) *p* = 0.274, WHO/IADPSG 12/116 (10%) vs. 12/117 (10%) RR (95% CI) 1.0 (0.4–2.2) *p* = 0.982, and NICE UK 19/116 (16%) vs. 13/117 (11%) RR (95% CI) 1.5 (0.8–2.8) *p* = 0.243 (Supplementary table S1). There was no significant difference across trial arms.

### Major harm

There were no significant harms or unintended effects attributable to eye-masks and earplugs in our trial.

## Discussion

In our proof-of-concept randomized trial with the intervention of EMEP in sleep-deprived pregnancy and primarily focused on glucose levels at OGTT and night sleep duration by actigraphy, fasting and 2-hour OGTT levels and night sleep duration were not significantly different.

Short sleep duration increases risk of developing GDM from meta-analysis data [9] Recent studies however show inconsistent relationship between self-reported short sleep and GDM [29] and short sleep duration is not an independent risk factor for GDM [30]. Our primary outcomes findings were self-consistent if EMEP was meant to drive glucose metabolism through sleep improvement.

EMEP in the home bedroom in late pregnancy has been shown in studies from our center to increase night sleep duration with actigraphy and when self-reported, by 23–25 minutes [15] over the baseline duration. Although this trial did not find a significant increase in actigraphy-derived night sleep duration but on post hoc analysis, change in self-reported night sleep duration (for which pre-trial and after intervention data were available) was modestly but significantly increased (by mean difference 0.2 h) with EMEP. Interestingly with regard to the potential utility of eye masks, light exposure before bedtime in pregnancy increases risk of developing GDM [31] and evening blue light exposure increases maternal fasting glucose [32]. Sleep intervention specific to pregnancy for patients with GDM was feasible in the context of typical care [25].

The absence of a significant increase in actigraphy-derived sleep duration compared with earlier trials [15–17] may be attributed to differences in participant characteristics and adherence to the intervention. In this study, participants exhibited less severe baseline sleep deprivation (median 5.8 h vs. 4.6 and 5.0 h in previous studies) and were at an earlier gestational stage (< 34 weeks vs. 34–36 weeks previously), which may have limited the potential for measurable improvement with EMEP. Furthermore, the > 10% dropout rate, largely due to non-compliance with nightly EMEP use and actigraphy wearing, could have attenuated the observed effect.

Our findings demonstrated that secondary outcomes of WASO, sleep efficiency, and sleep quality were not different. This is in keeping with the previous finding that no associations were demonstrated between the sleep quality measures (WASO, sleep fragmentation) and GDM [26] However, several other studies have demonstrated that impaired sleep quality such as increased sleep fragmentation, longer sleep latency, or frequent awakenings is independently associated with a higher risk of GDM [33, 34] Poor subjective sleep quality measured by the PSQI has been linked to elevated fasting glucose and insulin resistance, even after adjusting for sleep duration and BMI [35] In a large prospective cohort study, women reporting poor sleep quality during early pregnancy had significantly greater odds of developing GDM compared with those reporting good sleep quality [36] Similarly, actigraphy-based studies have shown that reduced sleep efficiency and greater wake after sleep onset are associated with impaired glucose tolerance and higher postprandial glucose levels during pregnancy [37, 38] Collectively, these findings underscore the multifactorial nature of sleep–glucose interactions, suggesting that not only sleep duration but also the restorative quality and continuity of sleep play key roles in maternal glucose regulation.

### Research implications

GDM complicated with short sleep duration was associated with increased risk for child neurodevelopmental delay [39]. Gestational sleep deprivation may increase offspring’s adiposity and blood pressure [40]. Chrononutritional and sleep hygiene intervention can improve maternal glycemic control [41] Treating sleep problems and improving sleep behavior in pregnancy could potentially reduce the risk and burden of GDM We did not find EMEP to impact on glucose metabolism or actigraph-derived night sleep duration but EMEP was only used for one week in our trial. It is plausible that longer use of EMEP could produce better results.

### Strengths and limitations

The strengths were the randomized parallel group trial design for a proof-of-concept study, sample size calculated with recently obtained pre-trial local OGTT results, and conservative estimates using pilot trial data of EMEP on sleep duration [15]. Limitations of this study included significant drop outs (> 10%) mainly because of non-compliance to actigraph watch use and presumably also of EMEP use in that trial arm. Generalizability could be reduced with trial data from only a single center. We did not perform pre-trial actigraphy so we could not compare the post intervention against the baseline. Previous behavioral or sleep hygiene interventions [42] showing improvement in glucose metabolism generally ranged from 1 to 8 weeks in duration. Our trial used a one-week intervention for pragmatic feasibility within the short window before routine OGTT. It is plausible that the duration was insufficient to elicit measurable metabolic changes. Another limitation was that actigraphy data were limited to nocturnal sleep periods. Daytime naps were not quantified, so total 24-hour sleep duration could not be determined. This is important, as some participants with short night sleep may have compensated with daytime naps, potentially attenuating the observed between-group difference.

## Conclusion

One-week use of eye mask and earplugs as a sleep aid in sleep-deprived pregnancies does not decrease glucose levels at oral glucose test or extend actigraph-derived night sleep duration.

## Supplementary Information

Supplementary Material 1.

## Data Availability

The datasets used and analyzed during the current study are available from the corresponding author on reasonable request.
