# Supplementary material for: Eye-mask and earplugs as sleep aid on glucose metabolism in sleep-deprived pregnancy: a randomized controlled trial
Source: BMC Pregnancy Childbirth. 2026 Jun 26;26:1004. doi: 10.1186/s12884-026-09283-y (PMC13560261; doi:10.1186/s12884-026-09283-y)
Supplement: Supplementary file 1 [file 12884_2026_9283_MOESM1_ESM.docx]

**Supplementary Table S1:** Post hoc analyses of glucose levels at 75-g oral glucose tolerance test (OGTT) and diagnosis of gestational diabetes mellitus (GDM) using various criteria in participants with sleep-deprived pregnancy randomized to eye-mask and earplugs (EMEP) or no intervention (control) prior to OGTT

|  | EMEP use | No intervention | RR (95% CI) | P value |
| --- | --- | --- | --- | --- |
|  | n = 116 | n = 117 |  |  |
| Glucose levels at OGTT |  |  |  |  |
| **Fasting** |  |  |  |  |
| ≥5.1^a^ | 8 (6.9%) | 7 (6.0%) | 1.2 (0.4-3.1) | 0.776 |
| ≥5.6^b^ | 2 (1.7%) | 1 (0.9%) | 2.0 (0.2-21.9) | 0.622 |
| **2 Hours** |  |  |  |  |
| ≥7.8^c^ | 18 (15.5%) | 13 (11.1%) | 1.4 (0.7-2.7) | 0.322 |
| ≥8.5^d^ | 7 (6.0%) | 8 (6.8%) | 0.9 (0.3-2.4) | 0.803 |
|  |  |  |  |  |
| GDM^e^ |  |  |  |  |
| **Malaysia** | 22 (19%) | 16 (14%) | 1.4 (0.8-2.5) | 0.274 |
| **WHO/IADPSG** | 12 (10%) | 12 (10%) | 1.0 (0.4-2.2) | 0.982 |
| **NICE UK** | 19 (16%) | 13 (11%) | 1.5 (0.8-2.8) | 0.243 |

Data represented as number (%). Analyses was by Chi-Square test for nominal data (Fisher exact test used instead if > 20% cells have cell size < 4)

^a^ ≥5.1 represent abnormal fasting plasma glucose according to WHO, IADPSG & Malaysian guideline in mmol/L

^b^ ≥5.6 represent abnormal fasting plasma glucose according to NICE UK Guideline in mmol/L

^c^ ≥7.8 represent abnormal 2 hours plasma glucose according to NICE UK & Malaysian guideline in mmol/L

^d^ ≥8.5 represent abnormal 2 hours plasma glucose according to WHO & IADPSG guideline in mmol/L

^e^ Diagnosis of GDM was made if either one of the fasting or 2-hour level is abnormal
